# Division of the Muscles of the Eye in Certain Cases of Blindness

**Published:** 1842-04

**Authors:** 


*Division of the Muscles of the Eye in certain cases of Blindness.*—When the central portion of the cornea has become so opaque as to prevent the transmission of the rays of light through the pupil, remaining sound, we may displace the axis of the eye, by dividing one or more of its muscles, so as to induce a certain degree of squinting, and thus bring a transparent part of the cornea in the direct line of vision. Such an operation is much more simple, and greatly less hazardous, than any of the modes which have been proposed to form an artificial pupil.

M. Florent Cunier, a well known ophthalmic surgeon in Belgium, claims the merit of having first performed it.

A man, 25 years of age, presented himself on the 21st of June, at his ophthalmic institution, with a strabismus of the left eye, which had existed from infancy. When two years old, he had suffered from a purulent ophthalmia, which had caused the destruction of this eye, and had left on the cornea of the other a dense opacity, which covered nearly the outer two thirds of its surface; the inner third, which remained transparent, was almost quite concealed in the angle of the orbit, and was visible at those times only when the squint was made to cease. When this was done he could see near objects, by carrying them towards the nose, and then turning the eye as forcibly as possible outwardly. The anterior chamber of the cornea was normal, and the pupil was quite free, and readily contracted on exposure to light.

On the 30th of June I divided, says M. Cunier, the internal rectus muscle; and immediately the pupil occupied the centre of the orbit, and the squint vanished. The eye being, however, not sufficiently drawn outwardly to enable the patient to see objects conveniently, I denuded the sclerotic as far as the attachments of the superior and inferior recti, but without causing the slightest degree of squinting outwards. Founding my practice on the experiments made by myself and Mr. Duffin, I divided the inferior oblique muscle; this was no sooner done, than at once the eye was drawn outwards and somewhat upwards. The ecchymosed blood in the cutaneous wound was rapidly absorbed; but the healing of the conjunctival wound was rather tedious, in consequence of the very considerable retraction of the muco-serous membrane induced by the displacement of the eye-ball. Immediately after the operation, the patient was able to guide himself through my garden; and six days afterwards he came alone to my house.

He now sees so well that he can distinguish the smallest objects, when they are brought near his eye. What is remarkable is, that the pupil has become displaced in such a manner that it is now immediately opposite to the transparent portion of the cornea.

Case 2.—A middle-aged man, who had been blind for 12 years, was my second patient. The left eye was completely wasted; and only a part of the outer half of the right one remained transparent. By compressing the left eye, and turning the right one forcibly inwards, he could perceive the form of large objects. I divided the external rectus muscle, and denuded the sclerotic as far as the insertion of the superior and inferior recti; the eye was immediately drawn inwards so as to cause a squint in that direction, and I was gratified to find that the patient was able to see much more distinctly. Eight days afterwards, he could spread out in the market-place his wicker-baskets, which he made to earn a subsistence.

In the third case, in which the operation for forming an artificial pupil had previously been attempted, but without success, M. Cunier divided the external rectus; and, although the case was certainly a very unfavorable one, considerable improvement of the vision was effected.—*Gazette Medicale.*

Remarks.—This application of ocular myotomy is certainly one of the most ingenious and scientific that has been proposed. The operation for making an artificial pupil being always hazardous, and seldom successful, surgeons will gladly avail themselves of the practice suggested by their Belgian *confrere.—Medico-Chirurg. Rev., Jan.* 1842, *p.* 211.

